# Human Lipocalin-Type Prostaglandin D Synthase-Based Drug Delivery System for Poorly Water-Soluble Anti-Cancer Drug SN-38

**DOI:** 10.1371/journal.pone.0142206

**Published:** 2015-11-03

**Authors:** Masatoshi Nakatsuji, Haruka Inoue, Masaki Kohno, Mayu Saito, Syogo Tsuge, Shota Shimizu, Atsuko Ishida, Osamu Ishibashi, Takashi Inui

**Affiliations:** Department of Applied Life Sciences, Graduate School of Life and Environmental Sciences, Osaka Prefecture University, 1-1 Gakuen-cho, Naka-ku, Sakai, Osaka 599-8531, Japan; Academia Sinica, TAIWAN

## Abstract

Lipocalin-type prostaglandin D synthase (L-PGDS) is a member of the lipocalin superfamily, which is composed of secretory transporter proteins, and binds a wide variety of small hydrophobic molecules. Using this function, we have reported the feasibility of using L-PGDS as a novel drug delivery vehicle for poorly water-soluble drugs. In this study, we show the development of a drug delivery system using L-PGDS, one that enables the direct clinical use of 7-ethyl-10-hydroxy-camptothecin (SN-38), a poorly water-soluble anti-cancer drug. In the presence of 2 mM L-PGDS, the concentration of SN-38 in PBS increased 1,130-fold as compared with that in PBS. Calorimetric experiments revealed that L-PGDS bound SN-38 at a molecular ratio of 1:3 with a dissociation constant value of 60 μM. The results of an *in vitro* growth inhibition assay revealed that the SN-38/L-PGDS complexes showed high anti-tumor activity against 3 human cancer cell lines, i.e., Colo201, MDA-MB-231, and PC-3 with a potency similar to that of SN-38 used alone. The intravenous administration of SN-38/L-PGDS complexes to mice bearing Colo201 tumors showed a pronounced anti-tumor effect. Intestinal mucositis, which is one of the side effects of this drug, was not observed in mice administered SN-38/L-PGDS complexes. Taken together, L-PGDS enables the direct usage of SN-38 with reduced side effects.

## Introduction

Most compounds that exhibit anti-tumor activities are known to be water-insoluble and to have severe side effects on normal tissues and organs, thus limiting their efficacy and clinical use of them [1]. Some common approaches to improve the solubility of anti-cancer drugs are the chemical modification of drugs and the usage of solubilizers such as organic solvents, surfactants, lipids, cyclodextrin, and pH modifiers. However, the chemical modification of drugs decreases their potency in many cases. The usage of solubilizers is limited due to their toxicity and tendency to cause drug instability. Thus, drug delivery systems (DDSs) for poorly water-soluble anti-cancer drugs that make effective use of different types of nano-sized delivery vehicle such as liposomes, polymer micelles, and dendrimers, have been investigated intensely [2–5]. These established DDSs, however, have also encountered some problems associated with toxicity, immunogenicity, hemolysis, and thrombogenicity [6, 7]. Therefore, there is a pressing need for the development of a novel DDS for poorly water-soluble anti-cancer drugs; and thus much effort has been focused on enhancing the potency, improving the safety, and increasing the solubility of these drugs.

**Fig 1:**
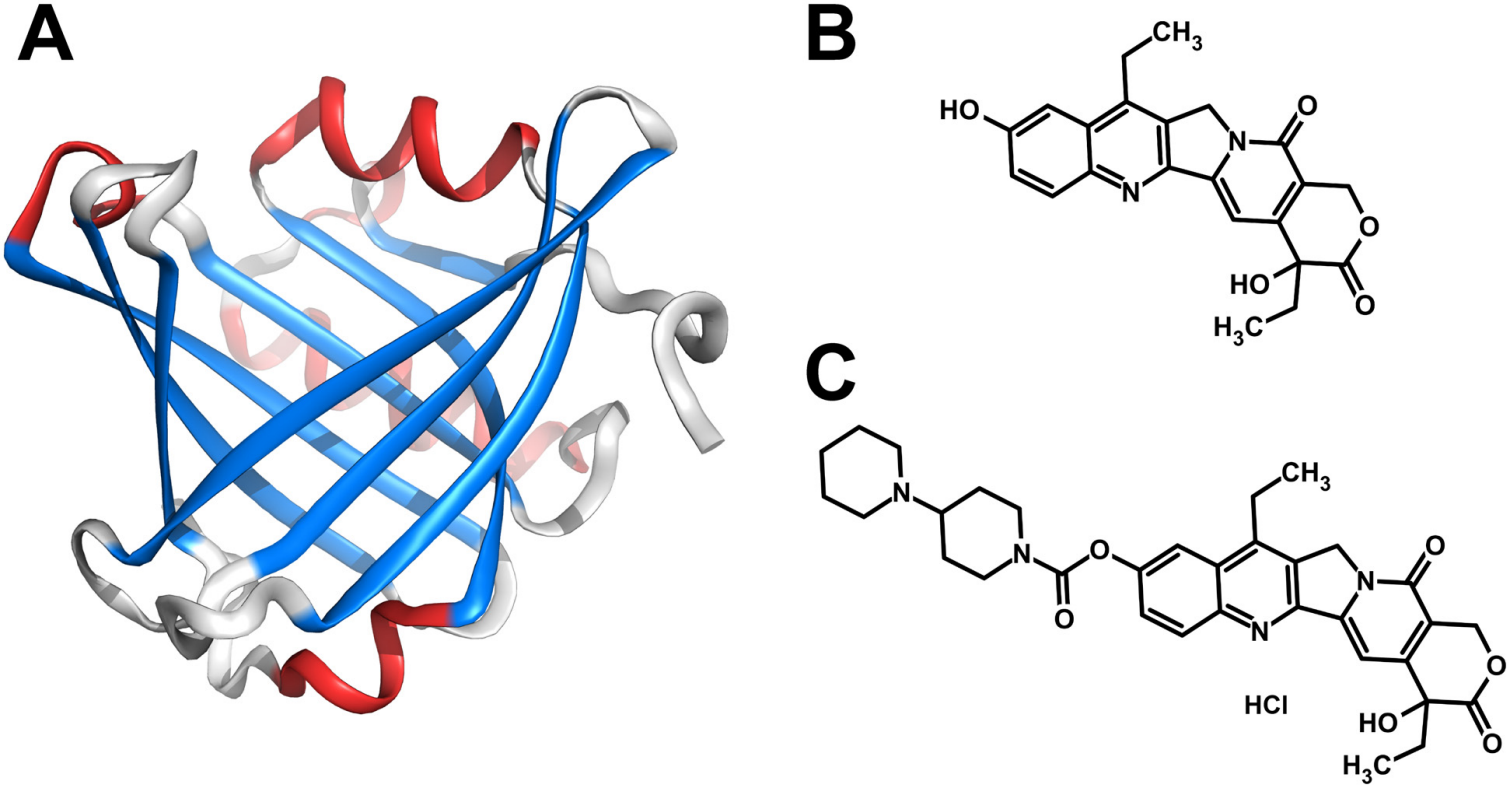
Structures of L-PGDS and compounds. (A) Crystal structure of human L-PGDS (molecular mass: 18777.7, PDB ID: 3O2Y). (B, C) Chemical structures of SN-38 (relative molecular mass: 392.4) and CPT-11 (relative molecular mass: 677.2).

We previously reported that a novel DDS using lipocalin-type prostaglandin D synthase (L-PGDS, Fig 1A), a member of the lipocalin family protein and a non-toxic and non-immunogenic molecule, could facilitate the pharmaceutical and clinical developments of poorly water-soluble compounds, such as diazepam and 6-nitro-7-sulfamoylbenzo[f] quinoxaline-2,3-dione, for use by either oral or intravenous administration [8]. L-PGDS is a multi-functional protein acting as a PGD_2_-producing enzyme [9], a scavenger of reactive oxygen species [10], and a secretory transporter protein for several small lipophilic molecules [11]. Moreover, we recently reported that L-PGDS acts as a scavenger of biliverdin, whose degradation products are involved in aneurysmal subarachnoid hemorrhage-induced vasospasm and neuronal cell death [12]. L-PGDS has a typical lipocalin fold that consists of an eight-stranded antiparallel β-barrel, and the interior of this barrel forms a hydrophobic cavity [13–15] that can bind a large variety of lipophilic ligands within it [11, 16].

SN-38, 7-ethyl-10-hydroxy-camptothecin (Fig 1B), is a semi-synthetic analogue of the anti-cancer alkaloid camptothecin that targets DNA topoisomerase I [17]. However, in spite of its potent anti-tumor activity, SN-38 has not been used directly in clinical practice due to its poor water solubility [18]. In addition, the lactone ring of SN-38 shows reversible pH-dependent hydrolysis, and at pH below 5.0, SN-38 exists in an active form with a close lactone ring in its structure, while it can be converted to an inactive carboxylated form at physiological pH by opening of the ring [19]. Thus, it is difficult to utilize SN-38 under a physiological condition. By contrast, irinotecan hydrochloride (CPT-11, Fig 1C), which is a water-soluble prodrug of SN-38, is used in combination with fluoropyrimidines as first-line therapy for patients with advanced colorectal cancer [20]. However, the chemical modification of SN-38 decreases its anti-tumor activity, leading to 1,000-fold less cytotoxic activity of CPT-11 compared with that of SN-38 against various cancer cell lines *in vitro* [21, 22]. Thus, the direct use of SN-38 as an active form using DDS might be great advantage for cancer treatment.

Here, we detail the development of a DDS using human L-PGDS, one that enabled the direct use of SN-38. We investigated the effect of L-PGDS on the solubility of SN-38, and examined the interaction between L-PGDS and SN-38 by using isothermal titration calorimetry (ITC) and small-angle X-ray scattering (SAXS). The cytotoxic activity of SN-38/L-PGDS complexes was evaluated by use of human colorectal, breast, and prostate cancer cell lines. Their anti-tumor activity was examined in the Colo201 human colorectal tumor xenograft model. To estimate the side effects of these complexes, we performed histopathological analysis and measured the expression levels of inflammatory cytokines in the small intestines. Finally, we performed anaphylaxis test to evaluate the immunogenic potency of L-PGDS. The results, taken together, demonstrated human L-PGDS to be a potent drug delivery vehicle for SN-38.

## Materials and Methods

### Materials

SN-38 was purchased from Tokyo Chemical Industry Co. Ltd. (Tokyo, Japan); and CPT-11, from Yakult Honsha Co., Ltd. (Tokyo, Japan).

### Purification of recombinant human L-PGDS

C65A/C167A (ε_280_ = 25,900 M^-1^ cm^-1^)-substituted L-PGDS was expressed as a glutathione *S*-transferase fusion protein in *Escherichia coli* BL21 (DE3; TOYOBO, Osaka, Japan) as described previously [16]. The fusion protein was bound to glutathione Sepharose 4B (GE Healthcare Bio-Sciences, Little Chalfont, UK) and incubated overnight with 165 units of thrombin to release the L-PGDS. The recombinant protein was further purified by gel filtration chromatography with HiLoad 26/600 Superdex 75 (GE Healthcare Bio-Sciences) in 5 mM Tris-HCl buffer (pH 8.0) and was then dialyzed against phosphate-buffered saline (PBS).

### Solubility measurements

An excess amount of SN-38 was added to PBS buffer (pH 7.4). The SN-38/PBS suspension was pre-incubated at 37°C for 30 min, and then mixed with an L-PGDS solution. This solution was then stirred at 37°C for 6 h and thereafter concentrated by using an Amicon Ultra Centrifugal Filter Device (Millipore Corporation, Bedford, MA). The absorption spectrum of the filtrate was obtained by use of a 1.0 cm-light-path quartz cuvette and DU800 spectrometer (Beckman Coulter, Pasadena, CA). The concentrations of SN-38 were determined spectroscopically based on the molar absorption coefficient of ε_380_ in DMSO for SN-38 = 20,985 M^-1^ cm^-1^.

### Isothermal titration calorimetry (ITC) measurements

Calorimetric experiments were performed with a MicroCal VP-ITC instrument (GE Healthcare Bio-Sciences), with the sample in PBS buffer (pH 7.4) containing 5% DMSO (v/v) at 37°C. L-PGDS (840 μM) in the injection syringe was reverse-titrated into 50 mM SN-38 in the cell. Titration experiments consisted of 50 injections spaced at 300–sec intervals. The injection volume was 2 or 5 μl for each injection, and the cell was continuously stirred at 286 rpm. The corresponding heat of dilution of L-PGDS titrated into the buffer was used to correct the data. The thermodynamic parameters were evaluated by using the one-set of independent binding sites model supplied by MicroCal Origin 7.0 software.

### Small-angle X-ray scattering (SAXS) measurements

SN-38/L-PGDS complexes in PBS buffer were passed through a filter to remove the insoluble compounds. The protein concentration of each sample was adjusted to suit SAXS experiments (3.0 mg/ml to 12.0 mg/ml). SAXS data were collected on beam line BL40B2 at SPring-8 (the synchrotron radiation facility, Hyogo, Japan), and all experimental procedures were the same as described previously [23]. Two-dimensionally recorded scattering patterns were converted to one-dimensional profiles by circular averaging. Contributions to scattering intensities from the solvent were eliminated from the raw data by subtracting the intensity curve obtained for the buffer solution. In order to calculate the radius of gyration for each protein, the scattering profile was analyzed by Guinier's approximation as described in the literature and in our previous report [23]. At each step, the interparticle interference and the effect of aggregation in the sample were carefully eliminated.

### Cell culture

Human colon cancer cell line Colo201 was purchased from Health Science Research Resources Bank (Osaka, Japan); and human breast cancer cell line MDA-MB-231, from American Type Culture Collection (Manassas, VA). Human prostate cancer cell line PC-3 was kindly provided by Prof. R. Yamaji (Osaka Prefecture University, Osaka, Japan). Colo201 and PC-3 cells were cultured in RPMI 1640 (Wako, Osaka, Japan) containing 10% fetal bovine serum (FBS) and 1% Antibiotic-Antimycotic (Life Technologies, Carlsbad, CA), whereas MDA-MB-231 cells were cultured in D-MEM (Wako) containing 10% FBS.

### *In vitro* growth inhibition assay

The effects of SN-38/L-PGDS complexes, SN-38, and CPT-11 on tumor cell growth were examined by performing the WST-8 assay (Nacalai Tesque, Kyoto, Japan). Colon adenocarcinoma-derived Colo201, breast adenocarcinoma-derived MDA-MB-231, and prostate adenocarcinoma-derived PC-3 cells were used in this assay. These cells were seeded into 96-well plates at densities of 5 × 10^3^ cells/well to 8 × 10^3^ cells/well. After a 24-h cultivation, the cells were treated with various concentrations of SN-38/L-PGDS complexes, SN-38 or CPT-11 for 48 h, and then the WST-8 solution was added the culture medium. Thereafter, the cells were incubated for 3 h at 37°C. The absorbance of formazan produced from WST-8 was measured at 450 nm by using a microplate reader, Model 680 (Bio-Rad Laboratories, Hercules, CA).

### Animal study

All mice used in this study were purchased from Japan SLC Inc. (Shizuoka, Japan). The mice were housed on a 12-h/12-h light-dark schedule with food and water available *ad libitum* for 1 week to allow recovery from the stress of transportation. All animal experimental procedures were approved by the Osaka Prefecture University Animal Care and Use Committee (Permit Number: 21–135). All surgeries were performed under isoflurane anesthesia, and all efforts were made to minimize suffering.

### *In vivo* growth inhibition assay

Five-week-old female BALB/c nude mice were injected subcutaneously in the right flank with 5 × 10^6^ Colo201 cells. When the tumor volume had reached 150 mm^3^, these mice were randomly divided into 6 test groups. SN-38/L-PGDS complexes at a dose of 1.0, 2.0 or 2.8 mg/kg/d, CPT-11 at a dose of 4.0 or 20 mg/kg/d, or PBS alone was administered intravenously every other day for 2 weeks. The length (a) and width (b) of the tumor and the body weight were measured every day, and the tumor volume was calculated as 1/2 (a × b^2^).

### Pathologic studies on small intestinal mucosa

PBS or SN-38/L-PGDS complexes at a dose of 2.8 mg/kg/d were administered intravenously to 5-week-old male ddY mice at the same dose schedule as those used in the growth inhibition assay *in vivo*. On day 15 after the first administration, the mice were sacrificed; and their small intestines were then isolated. The samples were fixed in 10% formalin, dehydrated, and embedded in paraffin, after which sections of 5-μm thickness were prepared and stained with hematoxylin-eosin.

### Real-time RT-PCR analysis

Total RNA was extracted from the small intestines by using RNAiso Plus (TaKaRa, Shiga, Japan) according to the manufacturer's protocol. The RNA was reverse-transcribed with the reagents of a PrimeScript RT reagent kit (TaKaRa). Real-time PCR analysis was performed by using THUNDERBIRD^®^ qPCR Mix (TOYOBO), and amplification was monitored with a Thermal Cycler Dice^®^ Real Time System II (TaKaRa). Primers used for this analysis are listed in S1 Table.

### Anaphylaxis test

Six-week-old male ddY mice were sensitized with either chicken ovalbumin (OVA, Sigma, Tokyo, Japan) or L-PGDS. OVA (2 mg/ml) or L-PGDS (2 mg/ml) was suspended in an equivalent amount of 13 mg/ml aluminum hydroxide (Sigma) as an adjuvant, and the suspension (100 μl) was administered subcutaneously into each mouse. After 14 days, either OVA or L-PGDS (1 mg/ml) was administered intravenously and the body temperature was then monitored by use of a thermofocus non-contact infrared thermometer, THERMOFOCUS (TECNIMED srl, Varese, Italy).

### Statistical analysis

Data were analyzed by using Student’s *t* test when the groups showed equal variances (F test) or with the Welch test when they showed unequal variances (F test). The results were considered significant at the 5% significance level (*P* < 0.05). All statistical tests were two sided.

## Results

### Improvement of the solubility of SN-38 by L-PGDS

**Fig 2:**
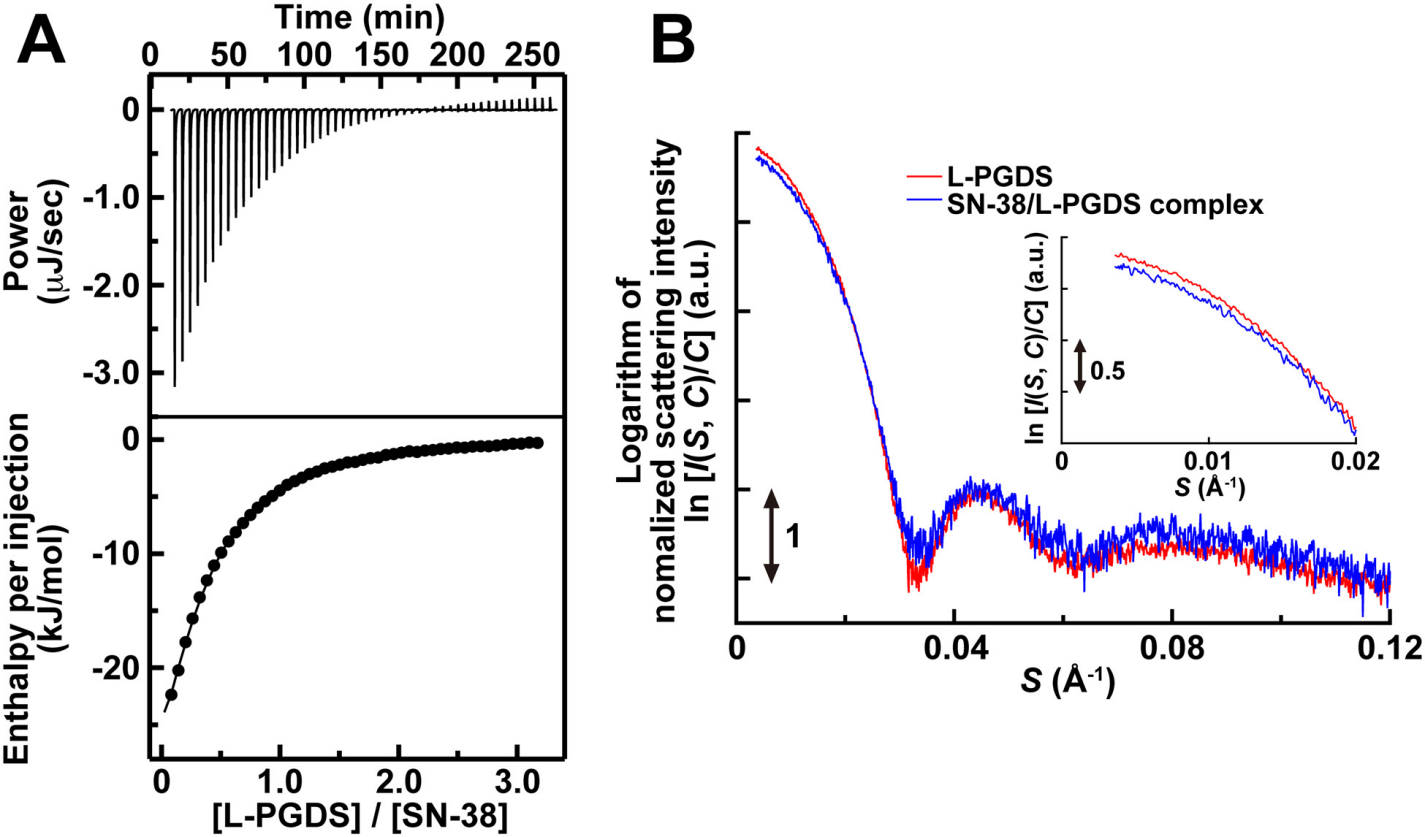
Characterization of the SN-38/L-PGDS complex. (A) Calorimetric titration of SN-38 with L-PGDS. L-PGDS in the injection syringe was reversely titrated to SN-38 in the cell. The upper panel shows the change in heat over time as L-PGDS was titrated into SN-38. The lower panel shows the normalized change in heat after subtracting reference data of L-PGDS injections into PBS. The one-set of independent binding sites model was used to fit the binding isotherms. (B) SAXS profiles of L-PGDS (red line) and SN-38/L-PGDS complex (blue line). These profiles were obtained by extrapolating all data at different concentrations (12, 9.0, 6.0, and 3.0 mg/ml) to the zero concentration. The logarithm of scattering intensity is shown as a function of the reciprocal vector (*S*). The inset shows the logarithm of scattering intensity in the small *S* region.

To examine the effect of L-PGDS on the solubility of SN-38, we measured the concentration of SN-38 in PBS with or without L-PGDS. SN-38 could be dissolved in PBS only up to 1.5 μM without L-PGDS. However, when 2 mM L-PGDS was added to PBS, the solubility of SN-38 increased up to 1,700 μM, which was 1,130-fold as compared with that in PBS. In addition, we carried out ITC and SAXS measurements to investigate the detailed binding mode of SN-38 to L-PGDS. In ITC measurements, by titrating L-PGDS to SN-38, we detected exothermic reactions, which indicated favorable enthalpy changes upon binding (Fig 2A, upper panel). After integrating each peak area, the integrated heat obtained for binding to SN-38 was plotted against the molar ratio ([L-PGDS]/[SN-38]) (Fig 2A, lower panel). The binding isotherm was fitted using the one-set of independent binding sites model, and the results revealed that L-PGDS formed a 1:3 complex with SN-38 with a dissociation constant (*K*_d_) value of SN-38 for L-PGDS of 60 ± 4.0 μM. The values of enthalpy change and entropy term for binding SN-38 to L-PGDS were -17 ± 0.13 and -8.5 ± 0.22 kJ/mol, respectively. These results showed that the interaction between L-PGDS and SN-38 was enthalpy-driven. Next, in SAXS measurements, the scattering intensity curves of L-PGDS and SN-38/L-PGDS complexes revealed that L-PGDS and SN-38/L-PGDS complexes were monodisperse without showing any aggregation up to the protein concentration of 640 μM (Fig 2B). These curves were similar, but obviously different only in the small-angle region (a reciprocal vector (*S*) < 0.02 Å^-1^, Fig 2b, inset). The values of radius of gyration of L-PGDS and SN-38/L-PGDS complex were calculated to be 18.1 ± 0.09 and 16.5 ± 0.20 Å, respectively. These results demonstrated that the structure of L-PGDS shrank when the protein bound SN-38.

### *In vitro* growth inhibition assay

**Fig 3:**
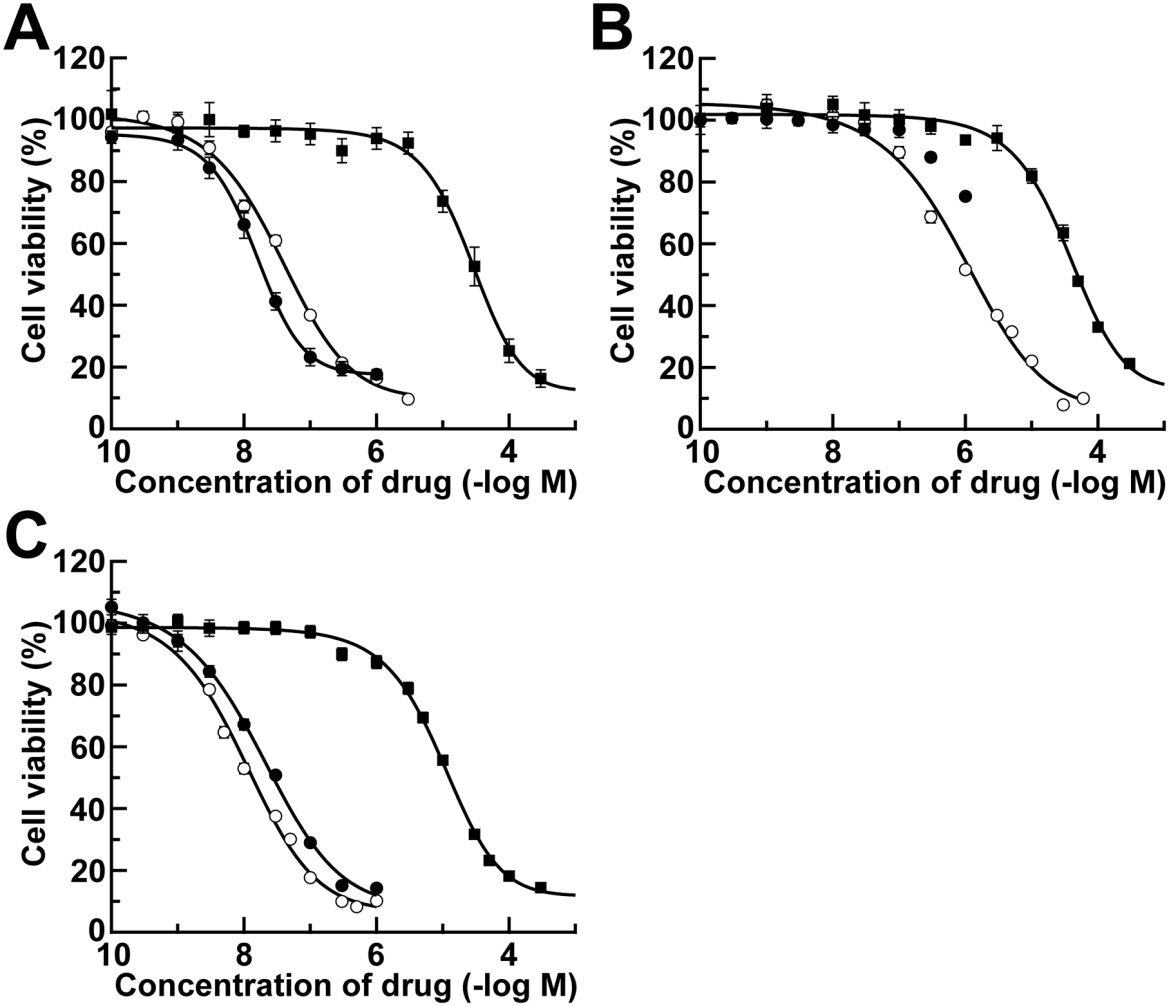
*In vitro* growth inhibition effect of SN-38/L-PGDS complex (○), SN-38 (●) and CPT-11 (■) on human colorectal cancer cell line Colo201 (A), breast cancer cell line MDA-MB-231 (B), and prostate cancer cell line PC-3 (C). The data are expressed as the mean ± SE. (*n* = 6).

In order to investigate the inhibitory effect of the SN-38/L-PGDS complex on cell growth *in vitro*, we measured the anti-tumor activity of SN-38/L-PGDS complexes against 3 human cancer cell lines, Colo201, MDA-MB-231, and PC-3, by performing WST-8 assays. SN-38/L-PGDS complexes, SN-38, and CPT-11 were separately dissolved in PBS and diluted with culture medium to the appropriate concentrations. All samples reduced the cell viability of all 3 cancer cell lines in a concentration-dependent manner (Fig 3). The calculated IC_50_ values are summarized in Table 1. The IC_50_ values of SN-38/L-PGDS complexes on the growth of Colo201, MDA-MB-231, and PC-3 cells were 35 ± 6.5, 900 ± 190 and 10 ± 1.5 nM, respectively, indicating that the complex was most potent against PC-3 cells. In contrast, those of CPT-11 on the growth of Colo201, MDA-MB-231, and PC-3 cells were 26 ± 4.1, 35 ± 5.2, and 9.8 ± 0.75 μM, respectively. Thus, the inhibitory effects of these SN-38/L-PGDS-complexes on the cell growth were 39- to 980-fold more potent than those of CPT-11. In addition, the IC_50_ values of SN-38 for the inhibition of growth of Colo201 and PC-3 cells were 15 ± 0.66 and 18 ± 2.4 nM, respectively, similar to those of the SN-38/L-PGDS complex. Thus, SN-38 showed high cytotoxic activity when it was possible to be solubilized in water. In the case of MDA-MB-231 cells, SN-38 at 1 μM, its maximum concentration in PBS, decreased the cell viability only to approximately 80%, and thus the IC_50_ value of SN-38 could not be obtained (Fig 3B). These results demonstrated that the SN-38/L-PGDS complex had a drug potency similar to that of SN-38 alone.

**Table 1:** Calculated IC_50_ values of SN-38/L-PGDS complexes, SN-38, and CPT-11 for inhibition of growth of Colo201, MDA-MB-231, and PC-3 cells.

Cell line	IC_50_		
	SN-38/L-PGDS complex (nM)	SN-38 (nM)	CPT-11 (μM)
Colo201	35 ± 6.5	15 ± 0.66	26 ± 4.1
MDA-MB-231	900 ± 190	N.D.*	35 ± 5.2
PC-3	10 ± 1.5	18 ± 2.4	9.8 ± 0.75

*N.D.: not determined

### *In vivo* growth inhibition assay

**Fig 4:**
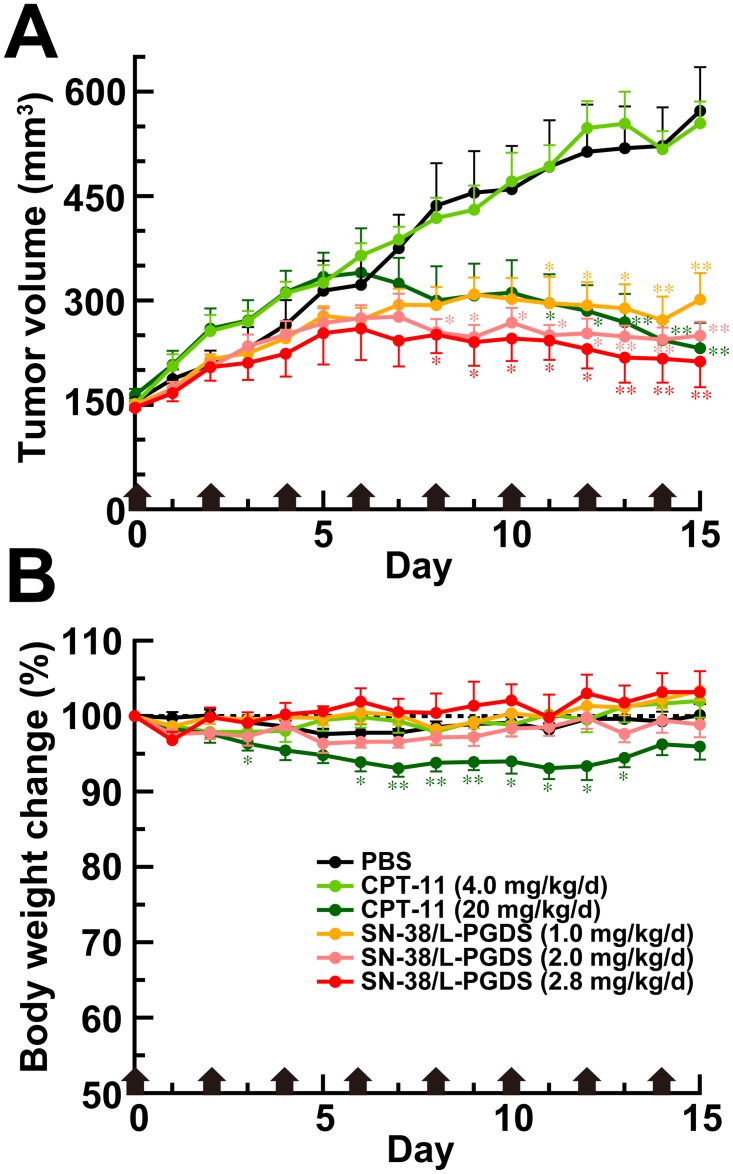
Anti-tumor effect of SN-38/L-PGDS complex. (A) Tumor volumes of PBS-, CPT-11-, and SN-38/L-PGDS complex-administered groups. Colo201 cells were inoculated subcutaneously into the right flank of mice. PBS, CPT-11 at a dose of 4.0 or 20 mg/kg/d, or SN-38/L-PGDS-complexes at a dose of 1.0, 2.0 or 2.8 mg/kg/d was administered intravenously once every other day for 2 weeks. * *P* < 0.05, ** *P* < 0.01 compared with PBS. (B) Body weight change of mice after each treatment. * *P* < 0.05, ** *P* < 0.01 compared with PBS. The error bars of the SN-38/L-PGDS complex-administered group at a dose of 2.8 mg/kg/d are expressed as the mean ± SE of 4 independent experiments; and those of the others, as the mean ± SE of 6 independent experiments.

We evaluated the anti-tumor activity of the SN-38/L-PGDS complexes in mice bearing Colo201 tumors. Fig 4A shows the effect of intravenous administrations of SN-38/L-PGDS complexes on the tumor in BALB/c nude mice bearing the tumor in their right flank. The PBS-administered group showed a progressive increase in tumor volume, reaching 572 ± 62.8 mm^3^ on day 15 after the first administration of PBS. In the CPT-11-administered group (4.0 mg/kg/d), the tumor volume increased rapidly in a day-dependent manner and reached 555 ± 31.3 mm^3^ on day 15 after the first administration of CPT-11, showing no anti-tumor activity. This pattern of day-dependent increase was similar to that for the PBS-treated mice used as a control. In contrast, although tumor growth in the mice treated with a high dose of CPT-11 (20 mg/kg/d) was almost the same as that observed in PBS-treated mice by day 6 after the first administration, the growth afterward was inhibited. The tumor volume was significantly different from that of the PBS-administered group after day 11, and reached 231 ± 36.5 mm^3^, which is 40.4% of that in PBS-administered group on day 15 after the first administration. On the other hand, although tumor growth in the mice treated with SN-38/L-PGDS complexes at a dose of 1.0 mg/kg/d was also almost the same as that observed in PBS- or CPT-11-treated mice by day 4 after the first administration, the growth afterward was inhibited. The tumor volume was significantly different from that of the PBS-administered group after day 11, and reached 301 ± 37.9 mm^3^, which is 52.6% of that in PBS-administered group on day 15 after the first administration. In addition, in the mice treated with SN-38/L-PGDS complexes at a dose of 2.0 mg/kg/d (which is an equimolar dose of CPT-11 at a dose of 4.0 mg/kg/d) or at 2.8 mg/kg/d, tumor regression was observed after day 4. After day 8, the tumor volume of the mice treated with SN-38/L-PGDS complexes at a dose of 2.0 mg/kg/d or 2.8 mg/kg/d was significantly lower than that seen in the PBS-administered group, reaching 249 ± 19.9 and 212 ± 36.9 mm^3^, respectively, which was 43.5 and 37.1%, respectively of that in the PBS-administered group on day 15 after the first administration. Thus, the SN-38/L-PGDS complex showed significant and dose-dependent anti-tumor activity. In SN-38-administered group (0.0029 mg/kg/d; its maximum concentration in PBS), however, the tumor volume increased in a day-dependent manner with tumor growth similar to that in the PBS- or CPT-11-administered group (4.0 mg/kg/d) and reached 536 ± 79.6 mm^3^ on day 15 after the first administration (data not shown). These results demonstrated that SN-38/PBS solution had any significant anti-tumor activity *in vivo*. Furthermore, in order to evaluate the side effects of the SN-38/L-PGDS complex, we recorded the body weight of the mice daily during the treatment (Fig 4B). In PBS-administered group, the body weight change was not observed. In CPT-11-administered group (20 mg/kg/d), however, the body weight loss was observed, and did not recover to normal level on day15 after the first administration. On the other hand, in SN-38/L-PGDS complexes-administered groups, the significant body weight loss was not observed within 15 days after the administration. These results, taken together, suggest that the intravenous administration of SN-38/L-PGDS complexes at a low dose showed higher anti-tumor activity than CPT-11.

### Evaluation of intestinal toxicity of the SN-38/L-PGDS complex

**Fig 5:**
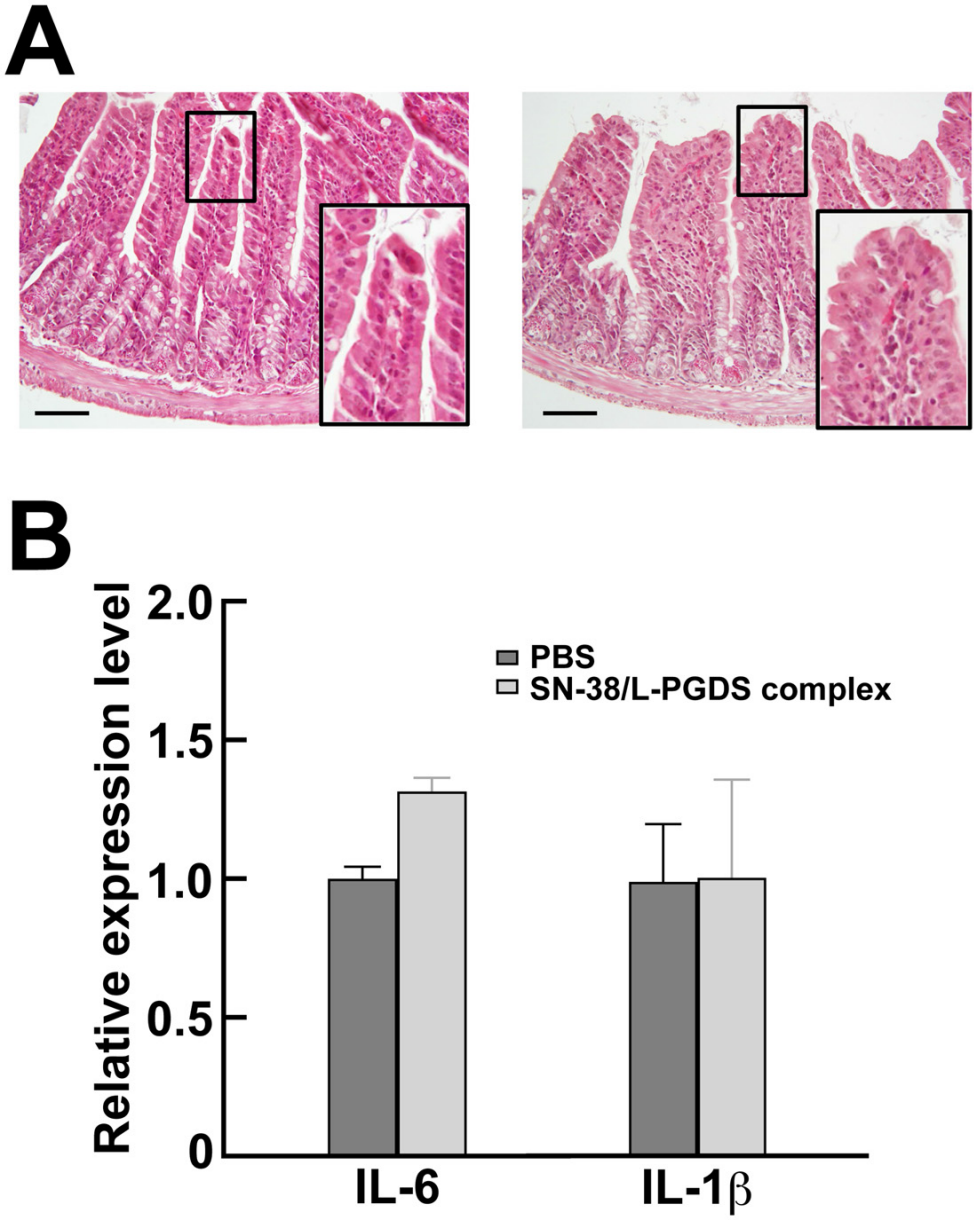
Effects of SN-38/L-PGDS complexes on the small intestines. (A) Histology of the ileal mucosa. Inset shows the villi architecture. Scale bar represents 100 μm. The left panel shows the ileal mucosa of the mice administered SN-38/L-PGDS complexes; and the right one, that of the mice administered PBS. The ileal mucosa in both groups was almost the same. (B) Expression levels of inflammatory cytokines in the ileal mucosa. Each bar represents the mean ± SD (*n* = 3).

It is well known that one of the major clinical side effects of CPT-11 is severe diarrhea [24, 25]. Several reports have shown that the administration of CPT-11 induces intestinal mucositis characterized by the loss of crypt architecture and the production of inflammatory cytokines [26, 27]. Thus, we investigated by histopathological analysis and the measurement of the expression levels of various inflammatory cytokines in the small intestines whether or not these side effects of CPT-11 were manifested by the SN-38/L-PGDS complex (Fig 5). The mice intravenously administered SN-38/L-PGDS complexes at a dose of 2.8 mg/kg/d by using the same dose schedule as used for the growth inhibition assay did not show any diarrhea. The histological observations of the intestinal mucosa demonstrated the preservation of the villi and crypt architecture, which was similar to that seen in the PBS-administered group (Fig 5A), thus indicating that the SN-38/L-PGDS complex did not induce intestinal lesions. In addition, the expression levels of interleukin-6 (IL-6) and interleukin-1β (IL-1β) in the intestines of the mice administered SN-38/L-PGDS complexes were unchanged compared with those for the mice administered PBS (1.3- and 1.0-fold, respectively; Fig 5B). On the other hand, in the intestines of the mice administered lipopolysaccharide, as a positive control, the up-regulation of IL-6 and IL-1β was observed (data not shown). Thus, these results revealed that the administration of the SN-38/L-PGDS complex did not show any side effects such as intestinal mucositis.

### Evaluation of immunogenic potency of human L-PGDS

**Fig 6:**
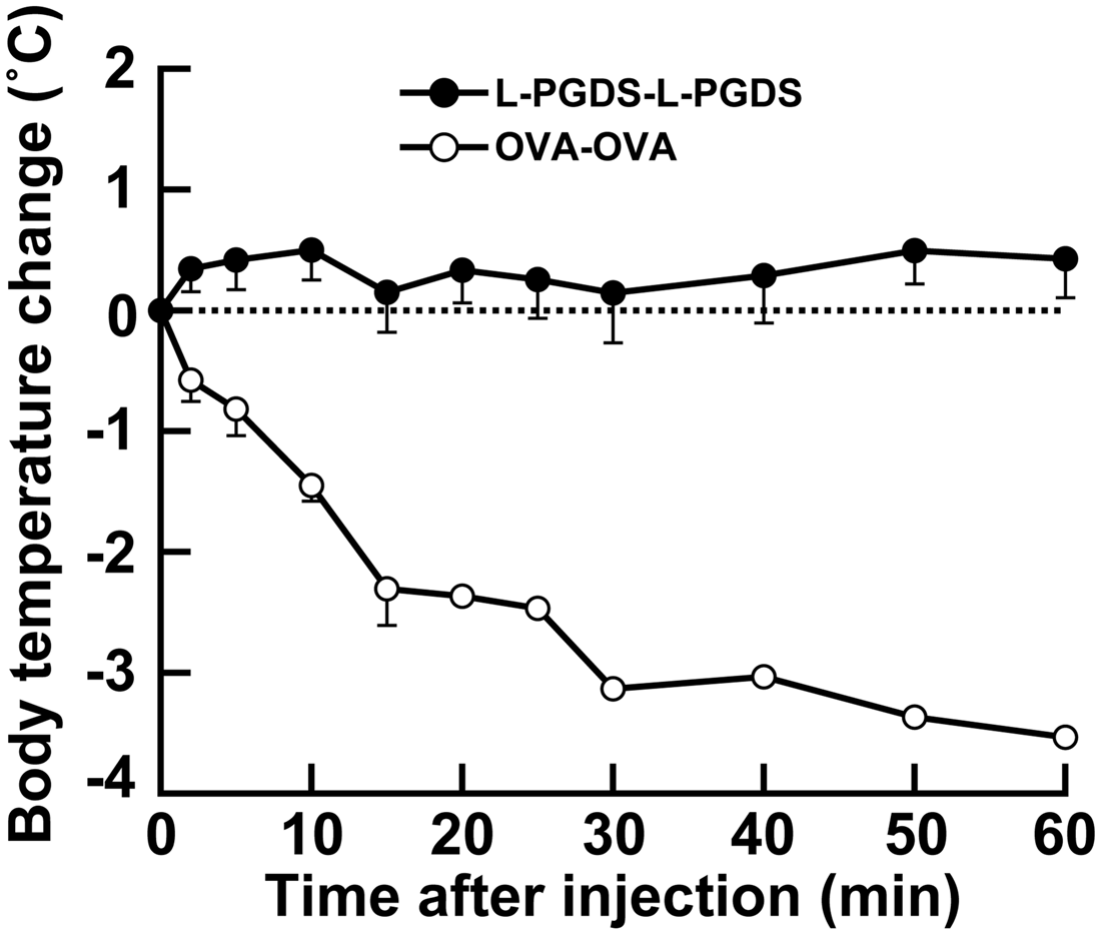
Systemic anaphylactic reaction in mice. Change in body temperature of mice. Mice were sensitized with OVA or L-PGDS, and then challenged with OVA (○) or L-PGDS (●). The body temperature was measured for 60 min. The data are expressed as the mean ± SE of 6 independent experiments.

Finally, we evaluated that whether human L-PGDS was immunogenic or not for mice (Fig 6). The body temperature of OVA-sensitized mice after the intravenous administration of OVA quickly fell. This decrease in body temperature did not recover for up to 60 min after the administration. On the other hand, the body temperature of human L-PGDS-sensitized mice after intravenous administration of human L-PGDS did not decrease. These results revealed that L-PGDS did not cause any anaphylaxis responses in these mice.

## Discussion

In this study, we showed the feasibility of using human L-PGDS as a novel drug delivery vehicle for SN-38, a poorly water-soluble anti-cancer drug. The solubility measurements revealed that the concentration of SN-38 was significantly improved in the presence of L-PGDS. Furthermore, the results of the *in vitro* growth inhibition assay revealed that the SN-38/L-PGDS complex showed a potent anti-tumor activity and that the complex formulation did not affect the drug potency of SN-38. In addition, the findings from the *in vivo* experiments revealed that the intravenous administration of SN-38/L-PGDS complexes resulted in a pronounced anti-tumor activity without the typical side effects of SN-38 such as intestinal mucositis. These results demonstrated that the complex formulation using human L-PGDS now makes it possible to use SN-38 for cancer treatment.

SN-38 has an efficacious anti-cancer activity against various cancer cell lines such as colorectal, lung, pancreatic, and ovarian cancer cell [21, 22], but it has not been used clinically due to its poor solubility in water and pharmaceutically acceptable solvents such as ethanol. Although CPT-11, a water-soluble prodrug of SN-38, is alternatively used for the treatment of colorectal cancer, the metabolic conversion rate is below 10% and depends on genetic variation in terms of carboxylesterase activity [28, 29]. Therefore, the administration of a high dose CPT-11 is necessary to achieve the expected therapeutic effect (Fig 4A) [30]. However, multiple dosing with a large amount of CPT-11 is limited owing to the severe side effects. The dose-limiting toxicity of CPT-11 administration is well known to result in the intestinal mucositis characterized by severe diarrhea [24, 25]. In the present study, we showed that the intravenous administration of SN-38/L-PGDS complexes at a low dose showed a pronounced anti-tumor activity as compared with that of CPT-11 (Fig 4A). In addition, histological analysis revealed that the intestinal mucosa of mice treated with SN-38/L-PGDS complexes at a dose of 2.8 mg/kg/d did not show shortened villi or the loss of crypt architecture (Fig 5A), which is one of the signs of intestinal mucositis. Furthermore, the mRNA levels of inflammatory cytokines such as IL-6 and IL-1β in the intestines of mice administered SN-38/L-PGDS complexes were similar to those of mice injected with PBS (Fig 5B). Thus, we demonstrated that the direct use of SN-38 as a complex with L-PGDS showed an anti-tumor activity without evoking undesirable side effects such as intestinal mucositis.

So far, various approaches to solubilize SN-38 have been reported, e.g., by using macromolecular prodrugs and nanomedicine formulations such as liposomes and polymeric micelles [18]. However, these methods involve multiple and complicated reactions; and the use of toxic organic solvents is required. For instance, EZN-2208, a water-soluble macromolecular prodrug of SN-38, is produced by complicated steps including overnight reactions [31]. A chlorin-core star-shaped block copolymer micelle, which is a nano-sized photosensitizing agent capable of encapsulating SN-38, was prepared by using a lyophilization-hydration method [32]. However, this lyophilization process is time-consuming; and furthermore, this process requires the use of organic solvent such as dimethyl sulfoxide. Thus the toxicity of residual organic solvents should be of concern. In our case, however, the formulation of SN-38/L-PGDS complexes could be achieved by a simple reaction, i.e., only mixing an SN-38 suspension with L-PGDS solution without any organic solvent. Furthermore, we have also demonstrated that the solid oral formulation of poorly water-soluble drug/L-PGDS complex can be simply achieved by using a spray-drying technique [33]. Therefore, the drug formulation using L-PGDS has a great advantage in terms of preparation time and safety. On the other hand, related to the stability of SN-38, much effort has been devoted to incorporate the lactone form of SN-38 into drug delivery vehicle [34], and protect the lactone ring of SN-38 from hydrolysis [35, 36] because the active lactone form is easily converted to inactive carboxyl form under the physiological condition. In the present study, we showed the high anti-tumor activity of SN-38/L-PGDS complex *in vivo*, suggesting that the active form of SN-38 reached into the tumor cells. In the preparation of SN-38/L-PGDS complex, almost 100% of SN-38 was calculated to be complexed with L-PGDS from the 3:1 binding stoichiometry and the *K*_d_ value of SN-38 for L-PGDS (*K*_d_ = 60 μM) by using the one-set of independent binding site model equations reported previously [37], while 82% of SN-38 was also calculated to be still formed complex under the equilibrium condition after the intravenous administration of the complex in mouse blood. By the docking simulation using AutoDock vina [38], it was estimated that the lactone form binds to L-PGDS with higher affinity (Δ*G* = -31.4 kJ/mol) than the carboxyl form (Δ*G* = -25.9 kJ/mol). Thus, SN-38 in the complex is present as the lactone form, and thereby it might reflect the high anti-tumor activity of SN-38/L-PGDS complex *in vivo*. In order to understand the precise form of SN-38 in the complex, however, we have to clarify the molecular structure of SN-38/L-PGDS complex by using NMR spectroscopy or X-ray crystallography.

The immunogenicity of a drug delivery vehicle may cause reduced efficacy and severe side effects by changing the pharmacokinetics and tissue distribution of the drug [39]. Most drug delivery vehicles are often coated with a polyethylene glycol (PEG) to evade the immune system and remain in circulation for a prolonged duration. However, it was recently reported that PEGylated vehicles lose their long-circulating property with multiple dosing due to the generation of anti-PEG antibody [40]. There are also many reports questioning the use of PEG in DDS’s [41, 42]. In the present study, we demonstrated that human L-PGDS did not cause any anaphylaxis responses in mice (Fig 6). This is explained by the highly identical (72%) amino acid sequences between human and mouse L-PGDS’s. Therefore, we consider that human L-PGDS would be a non-immunogenic molecule in humans and that L-PGDS would be a safer drug delivery vehicle than others.

In DDS-based cancer treatment, selective drug targeting is one of the most important issues. Although we demonstrated that the SN-38/L-PGDS complex had a more potent anti-tumor activity than an equimolar dose of CPT-11, it is unclear whether SN-38 was selectively delivered to the tumor tissue. Thus, it is necessary to investigate the tissue distribution of SN-38 and/or L-PGDS after the administration of SN-38/L-PGDS complexes. In addition, we are planning to confer the tumor-targeting property onto L-PGDS by introducing tumor-targeting peptides such as Arg-Gly-Asp (RGD) [43] and Asn-Gly-Arg (NGR) [44] motif into the structure of L-PGDS.

## Conclusions

In this study, we demonstrated that the concentration of SN-38 in PBS increased significantly by using L-PGDS without any organic solvent, and that the SN-38/L-PGDS complexes showed high anti-tumor activity both *in vitro* and *in vivo* with reduced side effects. Thus, we conclude that human L-PGDS is a novel and potent drug delivery vehicle for SN-38.

## Supporting Information

S1 Table Primer sequences used for RT-PCR. (DOCX)
