# Supplementary material for: Human Lipocalin-Type Prostaglandin D Synthase-Based Drug Delivery System for Poorly Water-Soluble Anti-Cancer Drug SN-38
Source: PLoS One. 2015 Nov 3;10(11):e0142206. doi: 10.1371/journal.pone.0142206 (PMC4631600; doi:10.1371/journal.pone.0142206)
Supplement: S1 Table — (DOCX) [file pone.0142206.s001.docx]

|  | Forward | Reverse |
| --- | --- | --- |
| IL-6 | 5’-ctggagtcacagaaggagtgg-3’ | 5’-ggtttgccgagtagatctcaa-3’ |
| IL-1β | 5’-gatcccaagcaatacccaaa-3’ | 5’-ggggaactctgcagactcaa-3’ |
